# Comparison of narrow-band imaging with autofluorescence imaging for endoscopic detection of squamous cell carcinoma of the tonsil

**DOI:** 10.1007/s00405-023-08111-9

**Published:** 2023-07-18

**Authors:** J. Syba, K. Trnkova, L. Dostalova, M. Votava, E. Lukesova, S. Novak, M. Kana, M. Tesarova, M. Zabrodsky, J. Plzak, P. Lukes

**Affiliations:** 1 Department of Otorhinolaryngology and Head and Neck Surgery, First Faculty of Medicine, Charles University, University Hospital Motol, Prague, Czech Republic; 2 Department of Otorhinolaryngology, Third Faculty of Medicine, Charles University, University Hospital Kralovske Vinohrady, Prague, Czech Republic

**Keywords:** Narrow band imaging, NBI, Autofluorescence, AFI, Tonsillar carcinoma, Oropharyngeal cancer

## Abstract

**Purpose:**

Early detection of mucosal neoplastic lesions is crucial for a patient’s prognosis. This has led to the development of effective optical endoscopic diagnostic methods such as narrow band imaging (NBI) and autofluorescence (AFI). Independent of each other, both of these methods were proven useful in the detection of mucosal neoplasias. There are limited reported data comparing both methods for oropharyngeal cancer diagnostics. The aim of the study was to compare NBI and AFI endoscopic visualization of signs in identifying tonsillar squamous cell carcinoma (SCC) and assessing its extent and to determine whether the score was related to the evaluator’s experience.

**Methods:**

Patients with tonsillar SCC underwent endoscopic pharyngeal examination using NBI and AFI. Fiftyseven video sequences of examinations of lesions proven to be SCC were evaluated by three reviewers. The accuracy of determination of lesion extent and visualization of its endoscopic signs of malignancy were evaluated.

**Results:**

Endoscopic visualization of tumour spread was significantly better using AFI than NBI (p = 0.0003). No significant difference was found between NBI and AFI in the visualization of endoscopic malignancy determining signs (p = 0.1405). No significant difference was found among the three reviewers in the visualization of tumour spread and for identifying malignancy-determining signs in NBI endoscopy or AFI endoscopy.

**Conclusions:**

The results show that AFI obtained better results for assessing the extent of tonsillar cancers than NBI. Both methods were proven to be equal in the visualization of endoscopic malignancy-determining signs. Both are useful even for less experienced evaluators.

## Introduction

The incidence of oropharyngeal squamous cell carcinoma (SCC) is increasing significantly in developed countries [1–3]. The UK has seen a doubling in incidence between 1990 and 2006. There was a further doubling in incidence between 2006 and 2010. Tobacco smoking and alcohol consumption are already well-known risk factors for the development of head and neck cancers. The increasing incidence of oropharyngeal SCC has been attributed to changes in social behaviour and mainly to the increase in the prevalence of the Human Papilloma Virus (HPV) [4–6].

It is usually difficult to detect the early stage of SCC, and diagnosis is often performed at a late stage. The extent of the large lesion is associated with a large surgical resection, larger postoperative morbidity and shortened survival time. The treatment of locally advanced oropharyngeal tumours often worsens swallowing and is associated with severe voice disorders [7]. Therefore, it is crucial to make an early diagnosis of head and neck SCC.

Examination procedures for cancer patients generally have two main goals: early detection and accurate determination of the extent of the disease. Oropharyngeal SCC can spread over the mucosal surface or submucosally. Submucosal spread can be detected by radiological imaging methods such as computed tomography (CT) or magnetic resonance imaging (MRI). Early stages of oropharyngeal SCC are typically relatively superficial and are well visible with NBI and AFI. CT and MRI can fail to display these superficial lesions [8–10]. For these reasons, basic clinical examination and endoscopy are the most important diagnostic techniques. Originally, the endoscopic examination consisted of only white light imaging, with limited imaging ability. In the last two decades, the development of endoscopic techniques has produced several new endoscopic optical imaging methods that have improved the possibilities of accurate and early detection of superficial mucosal lesions [10–15]. Of these methods, NBI and AFI are the most available in practice [16].

**Fig. 1:**
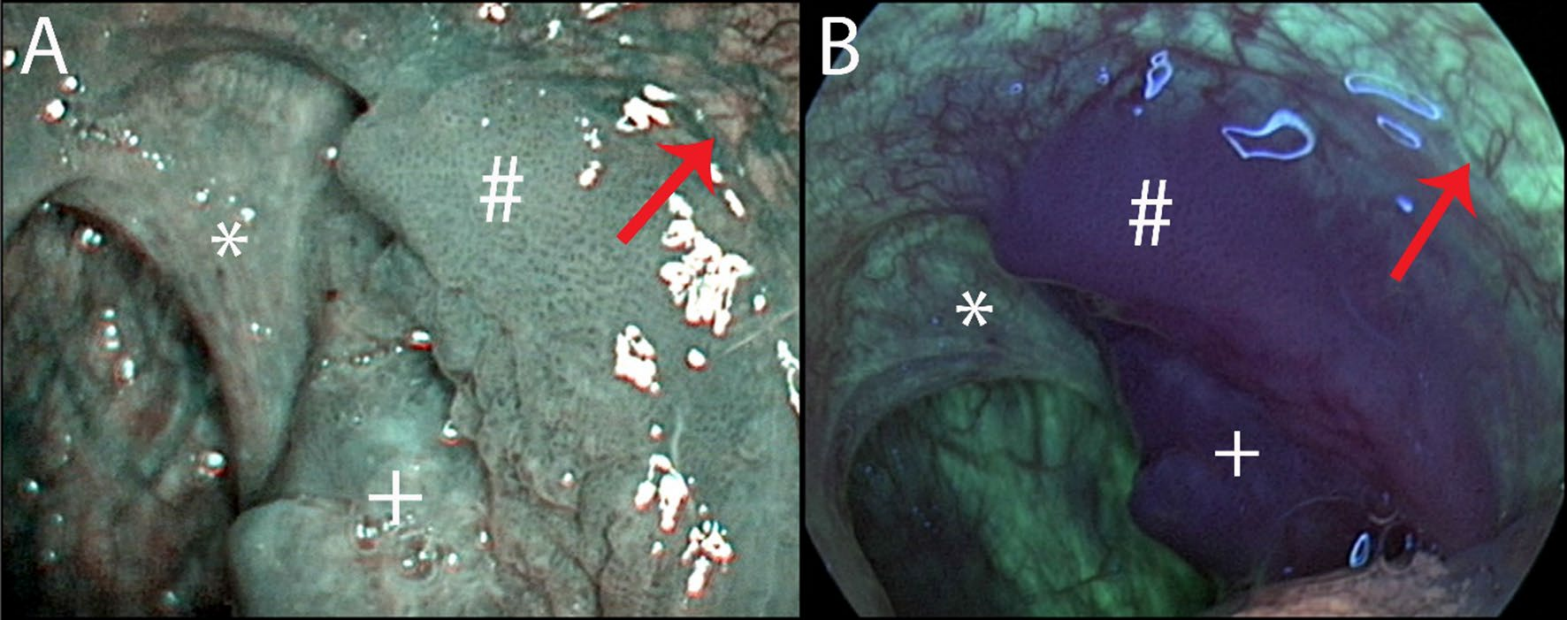
Cancer of the left tonsil in NBI (**A**) and AFI (**B**). Images evaluated as excellent both for visualisation of lesion extent and visualisation of signs that confirmed the malignant character of the lesion. Ulcerative lesion of the tonsil with pathological vascular changes in NBI and strong attenuation of AFI light ( +). Infiltration of anterior palatal arch clearly visible (#), posterior palatal arch with healthy mucosa (*). Healthy mucosa of the anterior palatal arch (red arrow)

**Fig. 2:**
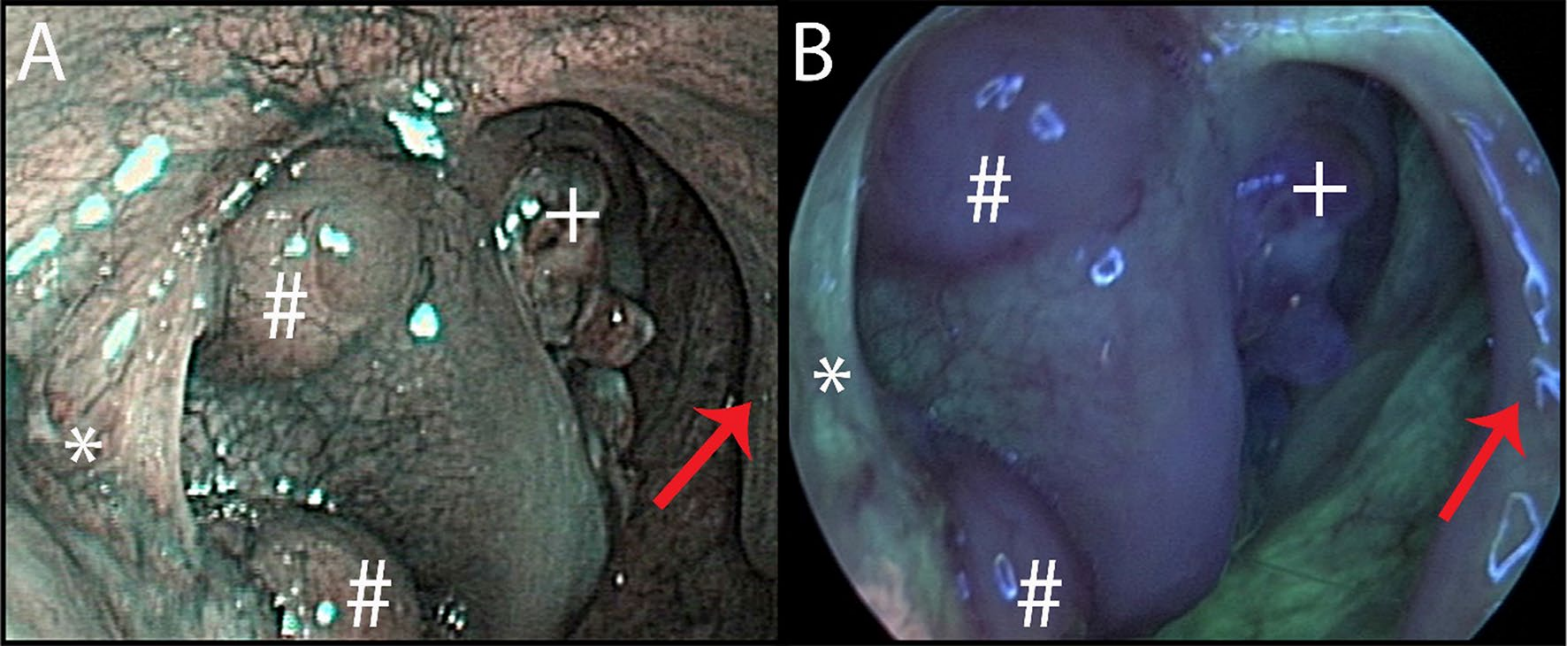
Cancer of the right tonsil in NBI (**A**) and AFI (**B**). Images evaluated as excellent both for visualisation of lesion extent in NBI and AFI and fair in NBI and excellent in AFI for visualisation of signs that confirmed the malignant character of the lesion. Ulcerative lesion of the tonsil with no pathological vascular changes in NBI and strong attenuation of AFI light ( +). Submucosal spread of tumour (#), anterior palatal arch with healthy mucosa (*), normal mucosa of the uvula (red arrow)

The NBI system contains a special image processor, and the light source of this system is equipped with narrowband filters that narrow the frequency range of emitted light to 400–430 nm (centered at 415 nm) and 525–555 nm (centered at 540 nm) bands. In contrast to red light, 415 nm wavelength light has less penetration and less scattering, thus enhancing image resolution. The blue filter is designed to correspond to the peak absorption spectrum of haemoglobin to enhance the image of capillary vessels (intraepithelial papillary capillary loops -IPCL) on the mucosal surface. Light at a wavelength of 540 nm highlights the submucosal vascular plexus. The reflection is captured by a charge-coupled device chip (CCD), and an image processor creates a composite pseudocolour image, which is displayed on a monitor, enabling NBI to enhance visualization of epithelial and vascular changes [17]. Using magnifying endoscopy with NBI increases the quality of vascular change visualization [18, 19]. Every mucosal neoplasia development and growth is accompanied by neoangiogenesis, which can be visualized as specific changes in IPCL (Figs. 1A and 2A). Changes in IPCL in the oropharynx could be classified according to Takano [20].

Fluorophores such as porphyrins, elastin, collagen and nicotinamide adenine dinucleotide (NADH) are commonly found in the mucosa [21]. These molecules have the ability to photoluminescent radiation, so-called autofluorescence. When illuminated with short-wavelength light, such as ultraviolet (UV) light, their electrons are excited to a higher energy level. They then return to their basic state by emitting heat and light of a longer wavelength than the light with which they were originally illuminated. This phenomenon can be displayed using a video endoscopic system with special filters. Because the concentration of fluorophores is different in healthy mucosa and in tumour-altered mucosa, there is a marked attenuation of autofluorescence in the case of tumour changes. Thickening of the epithelial layer and increased blood flow in pathological lesions may also contribute to this. Healthy mucosa is shown in bright green, and pathological mucosa is shown in magenta (Figs. 1B and 2B) [21]. In the oropharyngeal area, structures of palatal pillars and uvula usually show small attenuation of autofluorescence light emission in physiological status. The method enables better detection of small lesions that may not be visible under white light and allows accurate determination of the extent of mucosal malignancies [21].

Independent of each other, both methods have been proven to be very useful in the detection of mucosal neoplasias.

The aim of the study was to compare NBI and AFI endoscopic visualization of signs in identifying tonsillar squamous cell carcinoma and assessing its extent and determine whether the score was related to the evaluator’s experience.

## Materials and methods

Ninety patients with tonsillar lesions underwent in-office-based videoendoscopic examination at the Department of Otorhinolaryngology and Head and Neck Surgery, First Faculty of Medicine, Charles University and University Hospital Motol in 2014–2018. Some of them had the biopsy taken and SCC verified previously; in the other patients, we performed a biopsy right after the videoendoscopy. Patients with negative histology or with other than tonsillar SCC were excluded. Finally, 57 patients with tonsillar SCC were enrolled in this study. Video sequences were captured in all patients for further evaluation. First, routine white light endoscopic (WLE) examinations were performed; nevertheless, the WLE results were not considered in this study. Then, we performed NBI magnifying endoscopy and AFI endoscopy to assess the quality of the examination by evaluating the visualization of the lesion extent and by evaluating endoscopic signs that confirmed the malignant character of the lesion. We used the European Laryngological Society classification to evaluate IPCL changes in NBI [22]. We looked for perpendicular vascular changes to identify malignant structures.

For narrow-band imaging, we used OLYMPUS EXERA II and EXERA III systems with an OLYMPUS OTV-S7ProH-HD-12E camera head (OLYMPUS Medical Systems Co., Tokyo, Japan). For autofluorescence imaging, we used a Storz D-Light C (AF) System with a Tricam PDD camera head (Karl Storz, Tutlingen, Germany). For both NBI and AFI examinations, we used a Karl Storz 0° PDD telescope (8711 AP, 20 cm length, 10 mm diameter) (Karl Storz, Tutlingen, Germany). In the case of NBI, we used the camera with maximum close-up focus to achieve high magnification.

After the endoscopic examinations, one expert endoscopist (more than 10 years of experience) (P.L.), one experienced endoscopist (4 years of experience) (J.S.) and one resident (no experience with optical endoscopic diagnostic methods) (K.T.) retrospectively reviewed the NBI and AFI endoscopic video sequences. The quality of endoscopic visualizations of lesion extent was rated as being excellent, fair, or poor according to the visualizations and the possibility of confirming or negating tumorous infiltration of three anatomical sites as follows:

- Anterior palatal pillar and soft palate
- Base of the tongue
- Posterior palatal pillar and posterior pharyngeal wall.

An “excellent” visualization was defined as a video sequence in which the presence or absence of tumour spread was clearly visible in all three anatomical sites. A “fair” visualization was defined as a video sequence in which the presence or absence of tumour spread was clearly visible in two of three anatomical sites. A “poor” visualization was defined as a video sequence in which the presence or absence of tumour spread was clearly visible in only one or in no anatomical site.

For the evaluation of endoscopic signs that confirmed the malignant character of the lesion, the following criteria were set according to our experience and the literature: for NBI, the vascular structures were considered. The finding was considered “healthy mucosa” when no changed intrapapillary capillary loops (IPCL) and no surface disruption were visible. This finding was subsequently evaluated as “poor” visualization. In the case of visualization of changed mucosal tissue (ulceration, necrosis, fibrin, and keratosis) without changed IPCL, the finding was considered possibly malignant and evaluated as “fair”. In the case of visualization of changed mucosal tissue with changed IPCL, the finding was considered highly likely malignant and evaluated as “excellent”. For AFI, we compared the level of autofluorescence attenuation. Healthy tissue was considered to be bright green. This finding was subsequently evaluated as “poor” visualization. The attenuation of autofluorescence similar to that visible in the uvula and anterior pillars was considered possibly malignant and evaluated as “fair” visualization. If the attenuation was greater than that visible in the uvula and anterior pillars, we considered the finding to be highly likely malignant and evaluated it as “excellent” visualization.

We used the paired samples Wilcoxon test and Page test for ordered alternatives for statistical analysis of our results.

## Results

**Fig. 3:**
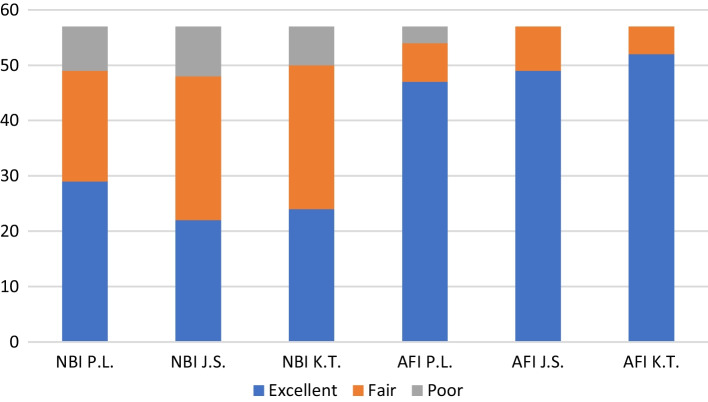
Visualization of tumour extent using NBI and AFI, P.L.:—expert, J.S.—experienced, and K.T.—resident endoscopist

Tonsillar squamous cell carcinoma was histologically confirmed in all 57 patients. NBI visualization of lesion extent was rated excellent in 29 patients, fair in 20 patients and poor in 8 patients by the expert endoscopist. It was rated excellent in 22 patients, fair in 26 patients, and poor in 9 patients by the experienced endoscopist and excellent in 24 patients, fair in 26 patients, and poor in 7 patients by the resident endoscopist. AFI visualization of lesion margins was rated excellent in 47 patients, fair in 7 patients and poor in 3 patients by the expert endoscopist. It was rated excellent in 49 patients, fair in 8 patients, and poor in no patients by the experienced endoscopist and excellent in 52 patients, fair in 5 patients, and poor in no patients by the resident endoscopist (Fig. 3). Endoscopic visualization of tumour spread was significantly better using AFI than NBI (paired samples Wilcoxon test: *p* = 0.0003). No significant difference was found among the three reviewers in the visualization of tumour spread for NBI endoscopy (Page test for ordered alternatives: *p* = 0.7129) and AFI endoscopy (Page test for ordered alternatives: *p* = 0.0800).

**Fig. 4:**
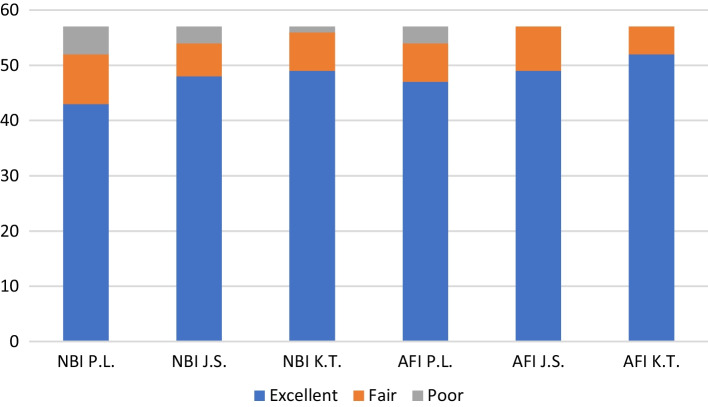
Endoscopic malignancy determining signs using NBI and AFI, P.L.:—expert, J.S.—experienced, and K.T.—resident endoscopist

NBI visualization of endoscopic malignancy determining signs was rated excellent in 43 patients, fair in 9 patients and poor in 5 patients by the expert endoscopist. It was rated excellent in 48 patients, fair in 6 patients, and poor in 3 patients by the experienced endoscopist and excellent in 49 patients, fair in 7 patients, and poor in one patient by the resident endoscopist. AFI visualization of endoscopic malignancy determining signs was rated excellent in 47 patients, fair in 7 patients and poor in 3 patients by the expert endoscopist. It was rated excellent in 49 patients, fair in 8 patients, and poor in no patients by the experienced endoscopist and excellent in 52 patients, fair in 5 patients, and poor in no patients by the resident endoscopist (Fig. 4). No significant difference was found between NBI and AFI visualization of endoscopic malignancy determining signs (paired samples Wilcoxon test: *p* = 0.1405). There was also no significant difference among the three reviewers for identifying malignant signs in NBI endoscopy (Page test for ordered alternatives: *p* = 0.0949) or AFI endoscopy (Page test for ordered alternatives: *p* = 0.1514).

## Discussion

When treating patients suffering from head and neck cancer**,** it is crucial to identify those tumours at an early stage to decrease the treatment morbidity and increase the survival rate. It is also very important to delineate the tumour borders precisely to perform safe R0 resections and to prevent the resection of more healthy tissue than necessary. Detection of early stages of oropharyngeal cancer is still relatively difficult because those lesions usually present minor morphological changes and could be left unidentified by conventional examination. Currently, various optical endoscopic imaging methods are increasingly used in practice for aerodigestive tract malignancy diagnostics. In the last 2 decades, papers on the use of autofluorescence and narrow-band imaging have been the most often published in the literature. Both NBI and AFI have already been proven to be useful methods for the detection of mucosal neoplasia of the upper aerodigestive tract [10, 21]. Recently, the use of autofluorescence seems to be on the decline, and the use of NBI is predominant. This may also be a consequence of the marketing intentions of the supplier companies. Based on the results obtained in this study, this may not be the most desirable trend. AFI has a longer history than NBI. The beginnings of the AFI date back to approximately 1920. Some scientific groups have tried to measure autofluorescence in healthy tissue and in tissue altered by a malignant tumour [23]. In the early 1960s, Ghadially et al. observed bright red fluorescence limited to the surface of ulcerated tumours [24]. Autofluorescence was first used in ENT practice in 1995. It was used by Kolli for the diagnosis of oropharyngeal tumours and by Harries for laryngeal tumours [25, 26]. In contrast, the idea of the NBI was first introduced in May 1999, and in December 1999 the first NBI image of living tissue was made using 415 nm light [17]. In 2005, the first-generation NBI investigation system was introduced [27]. While changes in vascularization in neoplasia are only visible as brown dots in normal (non-magnifying) NBI endoscopy, magnifying endoscopy, as previously described, allows for a more detailed observation of structural changes of mucosal vessels [15].

Both NBI and AFI have been reported to achieve very good results mainly in the diagnostics of laryngeal cancers. The good results are possibly caused by the presence of low-thickness epithelium in the area of interest [22, 28, 29]. In the oropharynx, three different types of epithelium with different thicknesses can be found [30]. The penetration depths of blue and green light are 170 μm and 240 μm, respectively. Due to these physical laws, the evaluation of some lesions in the oral cavity and oropharyngeal subsites, where the mean mucosal thickness measures up to 1300 μm, can be limited [31]. Because of these facts, the visibility of vascular changes in NBI endoscopy was significantly higher in the mouth floor, ventral tongue, soft palate, palatine tonsils, base of the tongue, epiglottis and hypopharynx than in other subsites of the oral cavity and hypopharynx [30]. For this reason, Takano et al. proposed a different classification for NBI findings in the oral cavity, which was proven to perform significantly better than other classifications [20]. In the meta-analysis by Ansari, NBI detection of oral and oropharyngeal malignant lesions achieved 76% sensitivity and 92% specificity [32]. In the publication by Piazza, the sensitivity and specificity of NBI were 89% and 85%, respectively, which were significantly better than the sensitivity and specificity of the WLI examination (78% and 73%, respectively) [33]. The sensitivity and specificity of AFI examination of oropharyngeal carcinoma were reported to be 95% and 95%, respectively [34].

In accordance with the aforementioned studies and according to our experience, the worst results are often obtained when examining the dorsal surface of the tongue, and good results are obtained when examining the tonsils. Therefore, in this study, we focused on tonsillar cancer examination, where both methods were expected to bring good results, and we wanted to determine whether one of them is superior to the other.

The goal of the endoscopic examination is not only to recognize a mucosal lesion but also to determine the degree of probability that this lesion is malignant. In the case of an already-diagnosed tumour, it is crucial to accurately determine the extent and spread of the given lesion as part of the pretreatment assessment. According to data in the literature, both AFI and NBI are highly effective in this role. To date, only a few studies have compared the effectiveness of both methods for different organ structures, including the oesophagus, lungs and larynx [18, 29, 35].

According to our information, a comparison of both methods for the examination of oropharyngeal malignancies has not yet been published in the literature. In this study, we did not focus on determining the sensitivity and specificity of both methods. All individuals had verified tonsillar cancer. However, we must keep in mind that both methods can yield false positive results. Especially inflammatory changes can be misdiagnosed as neoplasm. In NBI, vascular changes in the mucosa during inflammation can look similar to neoplastic changes at low magnification. However, the magnifying endoscopy used in this study is considered sensitive enough to distinguish such changes [15, 19]. Autofluorescence can also be attenuated due to increased blood flow in the mucosa during inflammation. For that reason, in this study we set the evaluation criteria according to the intensity of autofluorescence attenuation [36]. We focused on the comparison of the visualization of predetermined signs by both methods. Those signs were set separately for the AFI and for NBI. The correlation of endoscopic findings with histopathological results was not the aim of the study. The negative predictive value of NBI is reported 92 – 100% [37, 38] and negative predictive value of AFI 73–90% [34, 39]. Therefore when we did visualize normally looking mucosa, the site was considered without the tumour spread even without further histological confirmation. We focused on comparison whether this is better seen in NBI or AFI video endoscopy.

Our results show that both methods are equally effective in visualizing malignancy-determining signs of tonsillar cancer. Regarding the determination of the extent of the lesion, significantly better results were achieved using the AFI method. This is inconsistent with the results published by Japanese authors who tested both methods for oesophageal examination [18]. They concluded that NBI may be more effective than AFI for the visualization of oesophageal SCC. NBI endoscopy was also reported to be the better option for the diagnosis and differential diagnosis of laryngopharyngeal tumours by Ni et al. [10]. They reported comparable sensitivity of both methods but better specificity achieved by NBI. To date, no study addressing the use of AFI and NBI for oropharyngeal cancer is available for comparison with our results.

The possible explanation for the inconsistencies reported above may be the fact that we performed the examination transorally using a rigid endoscope. Larynx, hypopharynx and even oesophagus can be easily examined with the flexible video endoscope and a very short distance between the tip of the endoscope and the surface of mucosa can be easily achieved. This is crucial for the perfect visualisation of mucosal lesions especially in the case of NBI. However, this could be technically challenging during transoral examination of the oropharynx. On the other hand, AFI examination yields good results even from a larger distance from the surface of the mucosa. Moreover, in this study, three evaluators assessed prestored videos, however, patients were examined by several endoscopists of varying experience. This could be a drawback, as we assume that achieving good visualization in an NBI examination may be more difficult than in an AFI examination for the less experienced examinators. However, we do not yet have sufficient data to confirm this assumption. The limitation of our study is that the evaluation of the findings was based only on subjective assessment of video sequences. Nevertheless, according to the principles of the used methods, there is not yet a technology available that could quantify the findings objectively.

Our next goal was to determine whether the quality of video sequence assessment is influenced by the experience of the evaluator regardless of who performed the examination. The findings of the group of patients in the study were assessed by an evaluator with many years of experience, a moderately experienced physician and an inexperienced resident. There were no significant differences between the evaluators for either method. This means that the results of examination by both methods are very easy to evaluate even for less experienced physicians. Nevertheless, it could be more difficult to perform a perfect endoscopic examination in the case of the NBI method when it is necessary to get very close to the surface of the examined mucosa. Another limitation of this study is a small number of evaluators. We designed our experiment similarly to the study of Suzuki [18] which evaluated NBI and AFI for oesophageal cancer and was performed as a single-centre study with three evaluators. To remove this limitation a multicentre study should be organized, but we anticipate that it will not be easy to find many departments that have both endoscopic systems available.

## Conclusion

The results obtained in this study show that both NBI and AFI methods are suitable in the diagnosis of tonsillar squamous cell carcinomas, with slightly better results being obtained using AFI for assessment of the extent of the disease. Both methods were proven to be useful even for less experienced evaluators.

## Data Availability

Not applicable.
